# A Score Based on NfL and Glial Markers May Differentiate Between Relapsing–Remitting and Progressive MS Course

**DOI:** 10.3389/fneur.2020.00608

**Published:** 2020-07-16

**Authors:** André Huss, Markus Otto, Makbule Senel, Albert C. Ludolph, Ahmed Abdelhak, Hayrettin Tumani

**Affiliations:** ^1^Department of Neurology, University Hospital of Ulm, Ulm, Germany; ^2^Department of Neurology and Stroke, University Hospital of Tuebingen, Tübingen, Germany; ^3^Speciality Clinic of Neurology Dietenbronn, Schwendi, Germany

**Keywords:** multiple sclerosis, CSF, serum, glial markers, neurofilament light chain, progressive MS

## Abstract

**Background:** The diagnostic use of biomarkers in body fluids of multiple sclerosis (MS) patients allows the monitoring of different pathophysiological aspects of the disease. We previously reported elevated cerebrospinal fluid (CSF) and serum levels of glial fibrillary acidic protein (GFAP) but not neurofilament light chain (NfL) in progressive (PMS) compared to relapsing–remitting MS (RRMS) patients.

**Objectives:** We analyzed the glial marker chitinase-3-like protein 1 (CHI3L1) in the CSF and serum of PMS and RRMS patients. To capture the extent of glial processes in relation to axonal damage in each individual patient, we established a score based on CHI3L1, GFAP, and NfL and compared this score between RRMS and PMS patients and its association with the extended disability status scale (EDSS).

**Methods:** For this retrospective study, we included 86 MS patients (47 RRMS and 39 PMS) and 20 patients with other non-inflammatory neurological diseases (OND) as controls. NfL and GFAP levels were determined by the single-molecule array (Simoa). CHI3L1 levels were measured with classical enzyme-linked immunosorbent assay. A score was calculated based on glial to axonal markers (CHI3L1^*^GFAP/NfL, referred to as “Glia score”).

**Results:** CHI3L1 showed higher CSF levels in PMS vs. RRMS and controls (*p* < 0.001 and *p* < 0.0001, respectively), RMS vs. controls (*p* < 0.01), and higher serum levels for PMS vs. RRMS (*p* < 0.05). The Glia score was higher in the CSF of PMS compared to RRMS patients (*p* < 0.0001) and in the serum of PMS patients compared to RRMS (*p* < 0.01). Furthermore, the Glia score and CHI3L1 in serum but not in CSF correlated with the disability as determined by EDSS in the PMS group but not in the RRMS group (Spearman ρ = 0.46 and 0.45, *p* = 0.003 and 0.004, respectively).

**Discussion:** Our data indicate the involvement of glial mechanisms during the pathogenesis of PMS. Moreover, a calculated score may help to differentiate between PMS and RMS in the CSF and monitor disease progression in the serum of PMS patients.

## Introduction

The pathophysiology of multiple sclerosis (MS) is a complex interplay of B and T lymphocytes, demyelination, and axonal demise (1). Whereas, for relapsing–remitting MS (RRMS), the driving mechanism for disability in patients is supposed to be demyelination and the acute axonal damage (2). In progressive MS patients (PMS), the glial activation seems to be one of the major contributors to disability progression (3–5). Various glial processes are involved in MS, regardless of clinical subtype RRMS or PMS, like astrogliosis, microglial activation, scar formation, secretion of proinflammatory secretion of cytokines, and alteration of the metabolism of the neuroaxonal structures (6, 7).

The most extensively investigated marker for axonal damage is neurofilament light chain (NfL), which was shown to be elevated in the cerebrospinal fluid (CSF) of MS patients, correlating with MRI parameters of patients and being a potential prognostic biomarker (8–11). On the other hand, the concentration of glial fibrillary acidic protein (GFAP) is shown to be elevated in the CSF of PMS patients compared to RRMS patients (12) and correlate with the extent of contrast enhancement in RRMS patients (13). A further marker for glial activation, especially microglial activation, chitinase-3-like protein 1 (CHI3L1), was shown to be elevated in the CSF of MS patients compared to controls and a putative prognostic biomarker in patients with a clinically isolated syndrome (CIS) (14–18).

Highly sensitive detection methods as the single-molecule array allow the detection of brain-derived proteins in serum at low concentrations (19). Here, the determination of serum NfL is already well-established as a promising marker for prognosis and therapy efficacy (20–25) and as a possible additional endpoint for clinical trials (26). We could recently show that serum GFAP might be a more suitable marker for disease progression than serum NfL, as serum levels were higher in PMS patients compared to RRMS patients and correlated with extended disability status scale (EDSS) (27), also in a multicenter cohort (28). These findings were already independently confirmed by others (29).

Based on this first impression that glial processes might be an important driver of the disability in PMS patients, we analyzed CHI3L1 as an additional glial activation marker in the CSF and serum of PMS and RRMS patients. Together with the previously reported NfL and GFAP levels (27), we calculated a score based on those three markers for CSF and serum and compared its levels between RRMS and PMS patients and the correlation with EDSS.

## Materials and Methods

### Patient Selection

CSF and serum samples from 86 MS patients were collected at the Department of Neurology of the University Hospital Ulm between 2012 and 2017. Patients were characterized according to the revised McDonald criteria 2017 (30). Patients' disability status was determined by the EDSS. Relapses were defined as a focal neurological disturbance within the last 3 months lasting more than 24 h, without an alternate explanation.

Controls were selected from patients visiting the Department of Neurology of the University Hospital Ulm for a neurological examination but not showing abnormal MRI or CSF analysis (elevated cell count, total protein or albumin quotient, and no intrathecal immunoglobulin production). Diagnoses at the time of lumbar puncture were as follows in descending order: migraine or tension headache, functional disorders (e.g., non-organic hypoesthesia), and dissociative disorder. There were no statistical differences between the control group and the PMS or RRMS patient group concerning age and sex.

### CSF and Serum Sampling

CSF and serum samples were taken on the same day and processed according to the consensus protocol for CSF and serum collection and biobanking (31).

### CHI3L1 Measurements

CHI3L1 levels were determined using the Human Chitinase 3-like 1 Quantikine ELISA Kit (R&D Systems, Minneapolis, MN, USA). The assay was performed according to the manufacturer's instruction, and CSF was diluted 1:100 and serum 1:50. All samples were measured in duplicates, and intra-assay coefficients of variation (CVs) were <5% and interassay CV was <10%.

### Calculation of Glia Score

A biomarker score to represent glial processes compared to axonal damage for CSF and serum was calculated based on GFAP, CHI3L1, and NfL. GFAP and NfL values were published separately before (27) and used for this calculation. For the illustration of the extent of glial processes in relation to axonal damage, CHI3L1 and GFAP were placed in the numerator and NfL in the denominator of the equation. Thereby, the score is higher if glial processes are dominant and lower if axonal damage predominates.

The score was calculated for CSF and serum values as follows:

$$\frac{\text{GFAP}*\text{CHI3L1}}{\text{NfL}}$$

### Statistical Methods

All statistical tests were performed using GraphPad Prism 6 software (GraphPad Software Inc., La Jolla, CA, USA). The Shapiro–Wilk test was used to examine the distribution of the data. Mann–Whitney *U*-test and Kruskal–Wallis with Dunn's multiple comparison test was used to compare medians in skewed distributed datasets. Spearman's rho test was used to measure correlation. Receiver operating characteristics (ROC) analyses were performed to assess the discriminability of parameters between two groups. A *p* ≤ 0.05 was considered as statistically significant.

## Results

### Patient Characteristics

Paired serum and cerebrospinal fluid (CSF) samples of 86 MS patients (39 PMS patients−13 secondary PMS and 26 primary PMS; 47 RRMS patients) were analyzed as well as 20 other non-inflammatory neurological diseases {OND; median age, 44 years [interquartile range (IQR), 27–52]}. Seven RRMS patients received disease-modifying therapy (DMT); two received interferon-1-β (INF-β), three received Natalizumab, one Teriflunomide, and another one was on Alemtuzumab.

Three secondary PMS patients were on DMT (Natalizumab, INF-β, and Fingolimod). All clinical characteristics of the patients are summarized in Table 1.

**Table 1:** Patients' characteristics.

	***n*** = **86 MS patients**		
**Diagnosis**	**RRMS**	**PMS**	**Controls (OND)**
Number (*n*)	47 (44%)	39 (37%)	20 (100%)
Female (*n*)	29 (62%)	21 (54%)	13 (65%)
Age (years)	34 (27–47)	53 (47–59)	44 (27–51)
Recent relapse^*^	26 (55 %)	0	n/a
EDSS	2.0 (1.5–4.0)	6.0 (4.0–7.0)	n/a
DMT at LP	7	3	n/a
GFAP (pg/ml) (27)			
CSF	6836 (4,695–10,654)	11,131 (7,459–14,740)	6158 (2,425–8,064)
Serum	107 (74–141)	131 (98.6–224)	92.3 (57.2–140)
NfL (pg/ml) (27)			
CSF	1612 (871–3,205)	1,450 (1,045–2,340)	585 (358–835)
Serum	14.6 (9.2–26.8)	19.9 (13.9–28.4)	9.2 (6.0–12.3)
CHI3L1 (ng/ml)			
CSF	124 (88.3–162)	181.0 (151–254)	74.4 (47.5–96.1)
Serum	26.4 (21.4–35.4)	35.6 (23.8–96.3)	25.8 (21.7–43.2)

*Numbers are given as median and IQR or percentages in brackets*.

* *A recent relapse was defined as focal neurological disturbance lasting more than 24 h, without an alternate explanation*.

*RRMS, relapsing–remitting MS; PMS, progressive MS; PPMS, primary progressive MS; OND, other neurological diseases; EDSS, expanded disability status scale; DMT, disease-modifying treatment; LP, lumbar puncture*.

### CHI3L1 and Glia Score in the CSF and Serum of PMS and RRMS Patients

**Figure 1:**
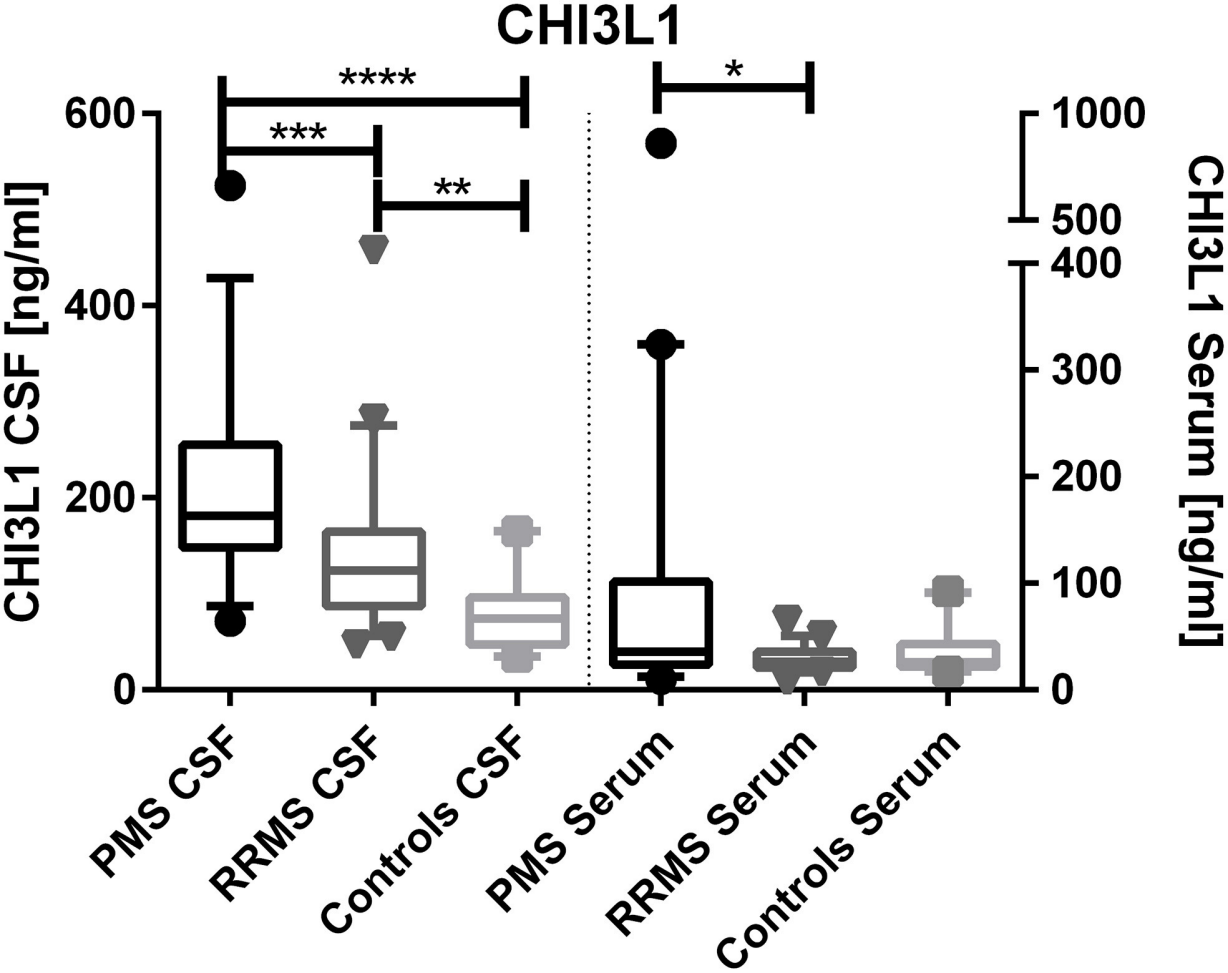
Chitinase-3-like protein 1 (CHI3L1) levels in cerebrospinal fluid (CSF) and serum of progressive multiple sclerosis (PMS) and relapsing–remitting MS (RRMS) patients. *p < 0.05; **p < 0.01; ***p < 0.001; ****p < 0.0001.

CHI3L1 levels were higher in PMS patients compared to RRMS in the CSF (median, 181 vs. 124 ng/ml, *p* < 0.0001) and in serum (median, 36 vs. 26 ng/ml, *p* = 0.0096) (see Figure 1).

**Figure 2:**
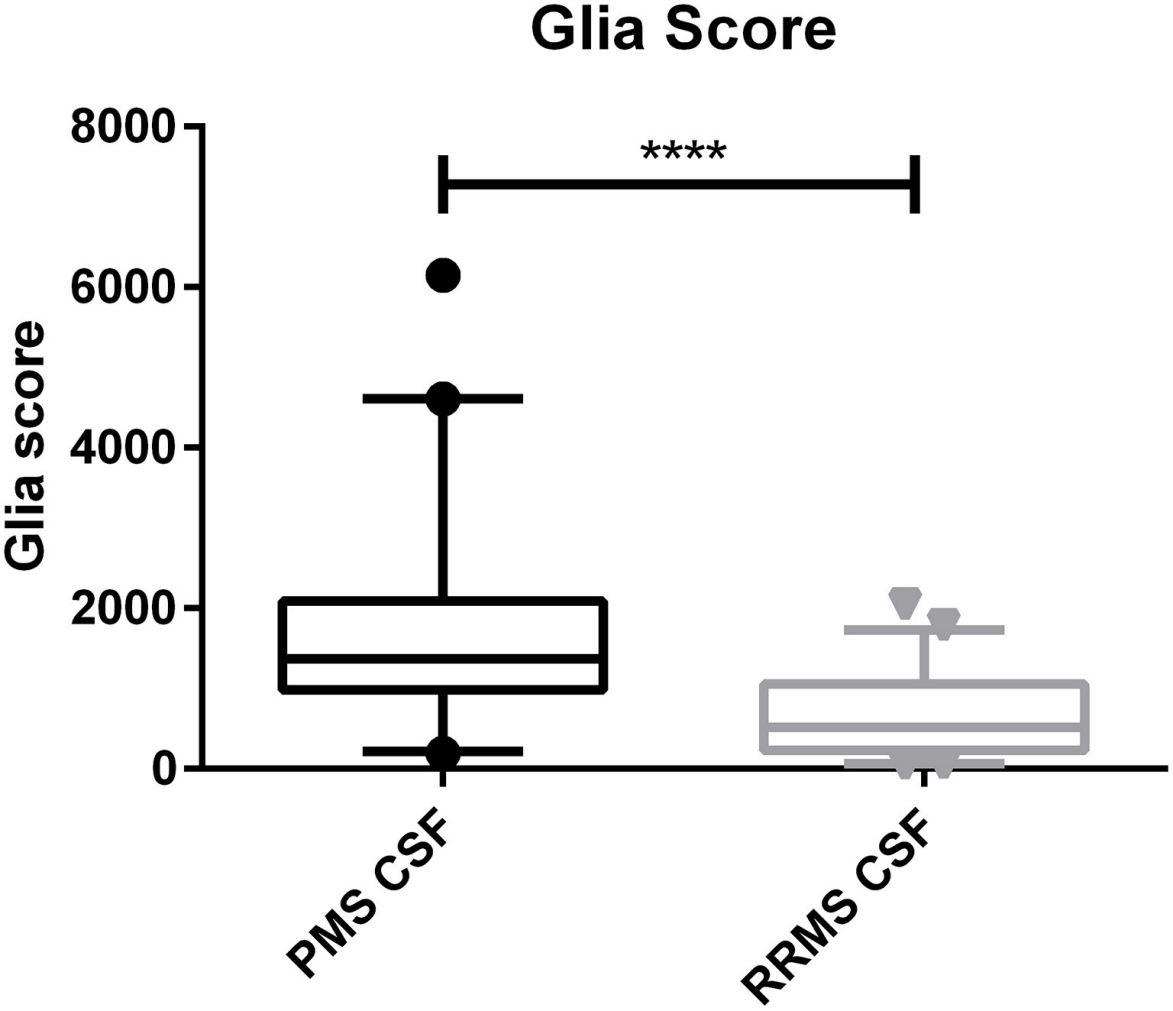
Glia score in the cerebrospinal fluid (CSF) of progressive multiple sclerosis (PMS) and relapsing–remitting MS (RRMS) patients. ****p < 0.0001.

**Figure 3:**
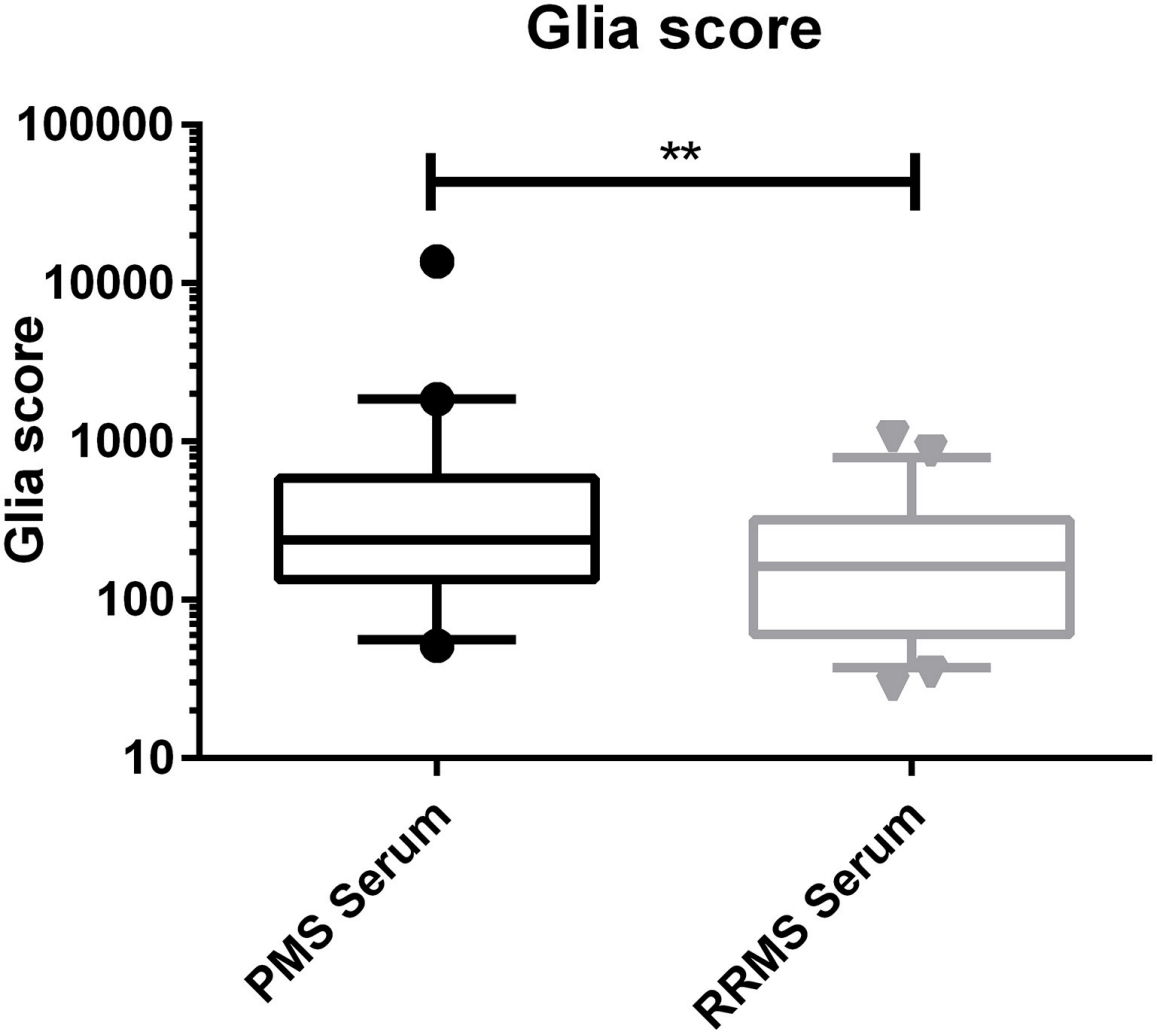
Glia score in the serum of progressive multiple sclerosis (PMS) and relapsing–remitting MS (RRMS) patients. **p < 0.01.

The calculated Glia score showed significantly higher levels in PMS patients compared to RRMS patients in the CSF (median, 1,369 vs. 519, *p* < 0.0001) and in serum (median, 239 vs. 163, *p* = 0.0032) (see Figures 2, 3). We could not observe a correlation between age and CHI3L1 or the Glia score in CSF and serum of PMS patients (Spearman ρ < 0.3) and only for age and the Glia score in RRMS patients (Spearman ρ = 0.53 for CSF and 0.38 for serum, *p* = 0.0002 and 0.008, respectively).

### ROC Analyses

**Figure 4:**
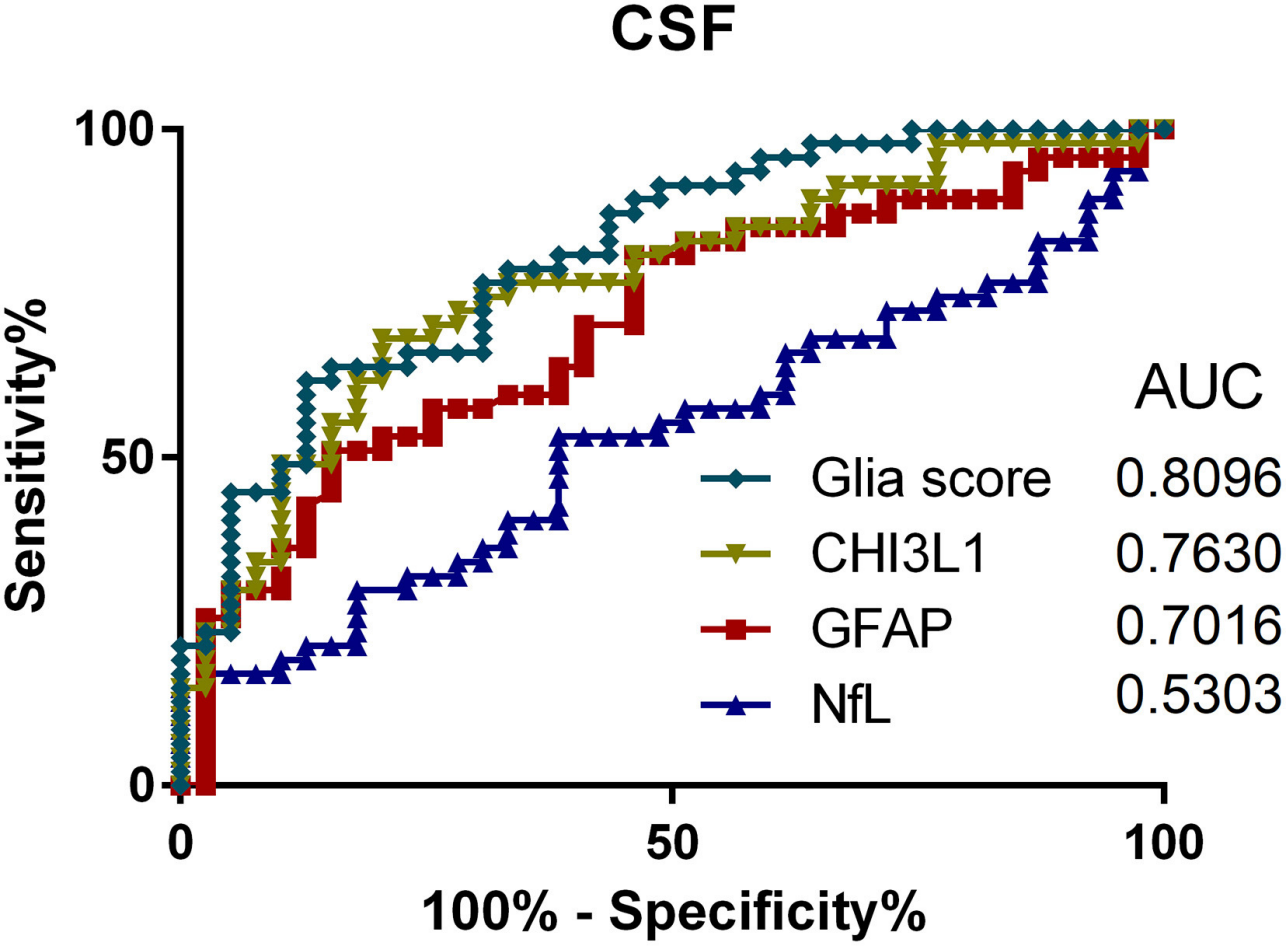
Receiver operating characteristics (ROC) analyses of Glia score (diamonds), CHI3L1 (triangle down), glial fibrillary acidic protein (GFAP) (square), and neurofilament light chain (NfL) (triangle up) in the cerebrospinal fluid (CSF) and for the comparison of progressive multiple sclerosis (PMS) vs. relapsing MS (RMS) patients. AUC, area under the curve.

**Figure 5:**
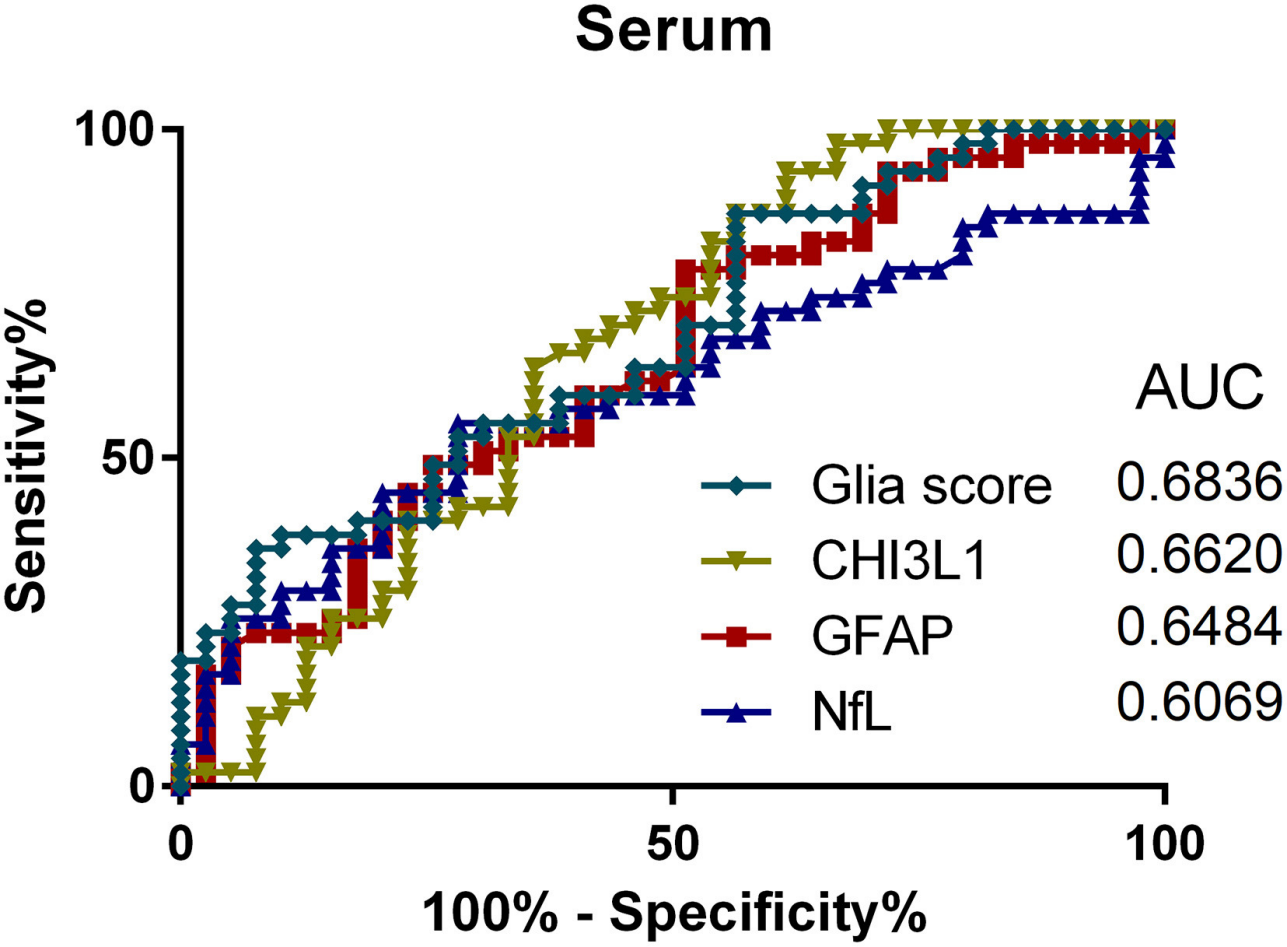
Receiver operating characteristics (ROC) analyses of Glia score (diamonds), CHI3L1 (triangle down), glial fibrillary acidic protein (GFAP) (square), and neurofilament light chain (NfL) (triangle up) in the serum and for the comparison of progressive multiple sclerosis (PMS) vs. relapsing MS (RMS) patients. AUC, area under the curve.

To compare the utility of the analyzed parameters regarding discrimination between PMS and RRMS patients, ROC analyses for CSF and serum were performed (see Figures 4, 5). Here, the Glia score showed the highest area under the curve (AUC) of 0.81, followed by CHI3L1 with 0.76, GFAP with 0.70, and NfL with 0.53 (Figure 4). Similarly, the Glia score in serum showed the highest AUC of 0.68 again, followed by CHI3L1 with 0.66, GFAP with 0.65, and NfL with 0.61.

### Correlation With EDSS

Correlation of CHI3L1 and Glia score in CSF and serum with the EDSS was calculated by Spearman correlation analysis, and Spearman rho and *p*-values are given in Table 2. Here, CHI3L1 and Glia score in serum showed a moderate correlation with the EDSS (ρ = 0.45 and 0.46, respectively) in PMS but not in RRMS patients.

**Table 2:** Spearman correlation of CHI3L1 and Glia score in CSF and serum of progressive MS (PMS) and relapsing–remitting MS Patients (RRMS).

**Spearman ρ**	**EDSS**	
	**PMS**	**RRMS**
CHI3L1 CSF	−0.05	0.14
CHI3L1 serum	0.45^**^	0.22
Glia score CSF	0.17	0.13
Glia score serum	0.46^**^	0.09

** *p < 0.01*.

## Discussion

Various factors contribute to the disability progression in multiple sclerosis, e.g., infiltration with different immune cells, glial cell activation, iron accumulation, and mitochondrial dysfunction. Those different pathophysiological aspects can occur in the same patient leading ultimately to demyelination and neuroaxonal demise. Nevertheless, some might overweight the others depending on the disease stage. The acute inflammatory reaction and the resulting demyelination and active axonal damage through the immune cells in the acute active plaques are apparent in RRMS and decrease over time (2, 32). On the other hand, the glial activation is a prominent driver of the disability in PMS through various mechanisms, including axonal dysfunction, and is not necessarily accompanied by remarkable acute neuroaxonal damage, as seen in the chronic inactive MS lesions (4, 5, 33–35). Based on this assumption, GFAP and CHI3L1 as markers of astrocytic and microglial activation showed higher levels in the CSF and serum of PMS patients compared to RRMS patients (11, 12, 18, 27, 29, 36–38). Moreover, GFAP, but not NfL, in serum correlated with the disease severity in PMS patients (27, 28).

In this work, we report similar findings using a marker of microglia activation in CSF and serum. In some scenarios, it might be challenging to determine the disease course solely based on the clinical presentation, like in patients with the first manifestation at older age or patients in the transitional phase between RRMS and SPMS. In our study, the proposed Glia score in the CSF might be a helpful tool to differentiate between RRMS and PMS patients, as it showed the highest area under the curve of all parameters.

There was a correlation between CHI3L1 and Glia score in serum and the EDSS, however only in PMS but not in RRMS patients. The same was shown for GFAP previously (27–29). Why these markers especially in serum but not in CSF show a correlation with EDSS remains a subject to be investigated. However, we hypothesize that glial processes that happen at the branches of astrocytes and astrocytic endfeet, which constitute a part of the blood–brain barrier and are in direct contact with blood vessels (39, 40), are well-reflected in the serum of those patients. Additionally, apoptosis and necrosis of astrocytes might release glial proteins as GFAP and CHI3L1 that are then drained via the glymphatic system into the blood (41). One example, therefore, is the very high levels of GFAP in the serum of neuromyelitis optica patients, where especially aquaporin-4 positive astrocytes are damaged (42, 43).

As this is an explorative study, these findings need to be confirmed in prospective, independent, and multicentric approaches with a higher number of patients including a comparison of active and inactive MS patients (44) and detailed MRI data. The suggested score is a first quite simplified approach to detect individual processes of neurodegeneration and glial activation in each patient. However, it needs further revision by a statistical weighting of single markers or the addition of further disease markers of MS.

Nevertheless, our data suggest that the glial markers GFAP and CHI3L1 might be a more suitable readout for disease progression and therapy response in PMS patients than NfL. Furthermore, we need to gain a deeper understanding of which of the various glial processes might be the driving mechanism of disease pathology in PMS, as neither GFAP nor CHI3L1 reflects specific glial processes.

## Data Availability Statement

The datasets generated for this study are available on request to the corresponding author.

## Ethics Statement

The studies involving human participants were reviewed and approved by Ethics committee University of Ulm, Ulm, Germany (approval number 20/10). The patients/participants provided their written informed consent to participate in this study.

## Author Contributions

MO, MS, and HT: study concept. AH and AA: data acquisition, data analysis, and interpretation. AH, AA, and HT: drafting of the manuscript. MO, HT, and AL: study supervision and critical revision. All authors: critically reviewed and approved the manuscript.

## Conflict of Interest

The authors declare that the research was conducted in the absence of any commercial or financial relationships that could be construed as a potential conflict of interest.
